# Using Arts‐Based Methods to Involve People Living in Tower Hamlets With Multiple Long‐Term Conditions in the Development of Artificial Intelligence Tools in Healthcare Research

**DOI:** 10.1111/hex.70621

**Published:** 2026-03-01

**Authors:** Elizabeth Remfry, Duncan J. Reynolds, Sylvia Morgado de Queiroz, Social Action for Health, Rohini Mathur, Michael R. Barnes, Alison Thomson, AI‐Multiply PPIE Group and AI‐Multiply

**Affiliations:** ^1^ William Harvey Research Institute Queen Mary University of London London UK; ^2^ Wolfson Institute of Population Health Queen Mary University of London London UK; ^3^ Oxford Brookes University Oxford UK; ^4^ Social Action for Health, Brady Arts Centre London UK; ^5^ Institute of Translational and Clinical Medicine, Faculty of Medical Sciences Newcastle University Newcastle upon Tyne UK

**Keywords:** artificial intelligence, arts‐based methods, participatory research, PPIE, public and patient involvement and engagement

## Abstract

**Background:**

Including public contributors in the development of artificial intelligence (AI) systems in healthcare research is growing, however, traditional methods of participation fail to engage people from minoritised groups. This work explores how we can utilise art‐based methods to involve the perspectives of those not previously included in AI development.

**Methods:**

We collaborated with a East London‐based organisation to involve people not previously included in research to contribute to a study on multiple long‐term conditions (MLTCs) and polypharmacy. Patient and public involvement and engagement (PPIE) contributors all had lived experience of MLTCs and represented a range of different ages, genders, socio‐demographic backgrounds and multilingual abilities. We ran a series of six workshops that used different visual arts methods; ceramics, collage, body mapping and AI‐generated images, to create research priorities and to inform AI development.

**Findings:**

The arts‐based methods served as a platform for communication which supported PPIE contributors to develop multiple research priorities, for example the impact of the lack of routine appointments on MLTCs. Through these workshops PPIE contributors also highlighted concepts that are important to consider during AI model development, such as utilising local housing data and considering bias. Visual images and art helped to facilitate different forms of communication, whilst being fun and engaging and provided a way to make abstract AI concepts more tangible whilst building AI literacy.

**Conclusions:**

Arts‐based methods were a useful tool to make involvement in research more accessible for under‐represented communities in the development of AI tools in healthcare research. There is a need for more inclusive participatory approaches as the use of AI in healthcare and research increases.

**Patient or Public Contribution:**

Working with staff and interpreters from a local community‐based charity, Social Action for Health, we invited 22 PPIE contributors from under‐represented communities in Tower Hamlets who had no previous experience of PPIE research. PPIE contributors developed the research priorities for a large academic consortia and helped create a community art exhibition to highlight their artwork. Additionally, two experienced PPIE contributors from the wider AI‐Multiply study assisted with the preparation of this manuscript.

## 1 Introduction

In the rapidly evolving landscape of artificial intelligence (AI) in healthcare, there is increasing evidence that these systems can perpetuate and exacerbate health inequalities in minoritised and disadvantaged groups [1, 2, 3]. Many AI models are referred to as ‘black‐box’ due to their mathematical complexity and difficulty to understand how they work behind the scenes [4]. The opaque nature of these algorithms makes it harder to interrogate the results and to understand where and how bias and discrimination occurs.

To mitigate algorithmic biases and create more equitable AI systems, there are growing calls to democratise AI by involving the *publics* throughout the development lifecycle [5]. The term *publics* encompasses the multitude of individuals to cover participant, citizen, public and in the case of healthcare, patients [6]. In this project, we use the term public and patient involvement and engagement (PPIE) contributor to refer to the publics involved.

The involvement of PPIE contributors in AI is under the assumption that through wider involvement and engagement this will result in more transparent systems and equitable outcomes [7]. However, there are limited examples of participatory AI work in practice within healthcare. How do we, as data scientists, work with the *publics* throughout the development of an algorithm? How do we broaden the range of perspectives, opinions and values that impact the development of AI systems? How can we ensure that participatory processes in AI development are inclusive and accessible to individuals with range of cultural, and experiential backgrounds, particularly those who do not speak English or have it as their first language? In this article, we explore participatory AI work in practice at the beginning of algorithm development in a health research setting. We examine the potential of using art‐based methods to ensure that under‐represented *publics* can get involved in algorithm building, we discuss the complexity that AI adds to participatory work, and finally how the involvement of PPIE contributors can inform algorithm building practices.

This work is situated in an academic research study focused on applying AI and machine learning methods to healthcare data [8]. The study, AI‐Multiply, uses “the collective expertise of patients, clinicians, researchers and AI to improve the care of people who live with many health conditions and medicines”. In this setting AI refers to AI methodologies, such as machine learning and deep learning, which are advanced statistical methods used to analyse and understand health data.

### 1.1 Participating in AI Research

While participatory and PPIE practices have a long and rich history in healthcare research [9, 10], it is still young in relation to AI, with some notable exceptions in the private sector [11], the European Union [12] and academia [6]. Participation can take many forms, including advisory groups, patient consultation, people's panels, co‐design and co‐production [10]. Examples of PPIE in AI research in healthcare can be found in: McInerney et al., [13] where PPIE contributors provided alternative perspectives on an AI system in acute care and Robinson [14] who tracks the involvement of a community redeveloping a flawed kidney transplant algorithm, highlighting how moral trade‐offs are made and influenced by patient contributors. Banerjee et al., [15] also discusses how the involvement of PPIE contributors in a project on severe mental illness pivoted the research direction to focus on explainable AI systems, and how patient involvement can also build trust in AI systems.

It is important to also highlight that all AI research in healthcare contains a level of passive participation. This passive participation takes place where patients generate data about their health, for example data that is recorded through routine checkups and appointments, which is stored in electronic healthcare records (EHR). This routine EHR data can then be extensively used to develop and train AI models without direct input from patients who agree to share their data for research purposes. This project aims to address this passivity, by inviting those who are generating healthcare data used in the AI‐Multiply study to have a say in how it is used and to actively participate in AI development. This approach actively challenges this passivity by encouraging democratic participation, which proposes that those who are affected by research, and in this case, whose data is used by the research, should have a say in it [16, 17].

In participatory research there is already a critique of the lack of diversity of contributors, manifesting as an overrepresentation of the ‘*usual suspects*’, predominately individuals who are white, retired, and middle class [18]. This overuse of *‘uncomplicated members’* is also apparent in AI research, where PPIE contributors tend to be well educated and from ethnic majority groups [6] and this overrepresentation may be intensified by the challenges around AI literacy. It is well documented that individuals in marginalised communities face disproportionate barriers to literacy and digital literary [19]. By relying on PPIE contributors who are already knowledgeable and comfortable talking about AI this may further intensify the lack of representation. If we want to ensure that we include those whose views, opinions and preferences are not already considered, we need to think differently about how researchers involve people in AI research, and foster understanding and engagement with AI concepts across different groups of society, in different contexts.

### 1.2 Arts‐Based Methods

In this study we explore the use of visual arts‐based methods to create meaningful opportunities for individuals who are often excluded from participation in AI research.

By using arts‐based methods this helps create inclusive ways to engage with PPIE contributors that move beyond traditional methods that rely heavily on written materials, academic presentations and qualitative research style focus group discussions. Arts‐based methods can be playful and fun, making participating in research more engaging and inviting [20]. Methods, such as collage, drawing and ceramics, are practical and hands on activities that use familiar materials that enable multiple ways of knowing [21, 22, 23]. Inherent to PPIE is the recognition that PPIE contributors bring differing forms of knowledge to the research process and it places equal importance on lived experiential knowledge and technical knowledge that researchers may hold. By creating art and visual images, this allows for the sharing of different types of knowledge held by different stakeholders and can also make tacit knowledge or ‘know‐how’, which is often difficult to verbalise, more explicit [21]. Both the process of creating and the final art piece provide points of communication and help to bring forward the everyday lived experiences of contributors. This can provide alternative ways to look at problems, and identify new issues that need to be addressed through academic research [24].

Arts‐based methods have been posited as a way to facilitate the involvement of seldom heard and under‐served contributors in research as they utilise existing skills and abilities [25]. The creation of art can also challenge the traditional power dynamics within research as they blur the lines between stakeholders enabling the sharing of different ideas [26] and the artwork created can also help to more effectively communicate lived experiences to researchers [27]. Previous research has also highlighted that arts‐based inquiry can lead to empowerment amongst PPIE contributors as it can help build confidence, develop skills and social networks, and in some cases lead to institutional or policy change which is bought about as a result of arts‐based inquiry [27].

A challenge with participatory approaches in AI research is the highly technical language of AI. However, visual arts‐based methods have been used to provide a platform for learning about complex concepts [28]. For example, recent projects have explored how art‐based methods can offer novel approaches to developing AI literacy, through co‐developing a song [29], the creation of artistic work to ‘make underlying systems visible’ to reveal AI pipelines [30] and co‐developing an animation around statistical methods [28, 31].

This project builds on these works exploring the use of arts‐based methods to facilitate PPIE in AI research. There were multiple aims to this project; (1) to explore the use of creative methods to involve under‐represented contributors in AI research, (2) to develop a list of priorities for research on MLTCs and taking multiple medications based on PPIE contributors feedback.

## 2 Materials and Methods

### 2.1 PPIE Contributors

A total of 22 contributors were invited by Social Action for Health, a community‐based health charity based in East London that supports people most affected by health inequalities [32]. PPIE contributors were invited through Social Action for Health's existing network and events, such as English language classes, parenting groups and men's mental health support groups. The requirements for PPIE contributors was the ability to attend workshops in person in East London, and have lived experience of multiple long‐term conditions and taking multiple medications. All 22 individuals were local to Tower Hamlets, a borough with the third highest poverty rate and premature death rate in London [19]. There was a mixture of genders, ages (ranging from 29 to 70+), languages spoken and ethnicities. Three contributors spoke English as their first language, 13 spoke English as an additional language, and six contributors did not speak English. Ethnicity was self‐identified, resulting in six ethnic groups, with the majority (68%) identifying as Bengali. Overall, the group broadly reflected the diversity of the local population of Tower Hamlets. Many of the group were new readers and learning to use digital technologies, such as laptops. Some had an understanding of AI, although the majority were not familiar with it and none of the contributors had prior PPIE experience.

The PPIE contributors were reimbursed for each workshop via vouchers for a local supermarket.

### 2.2 Research Team

The team consisted of ER, a data science PhD student from Queen Mary University of London; GC, a project manager at Social Action for Health; CD, CEO at Social Action for Health; RK, an interpreter from Social Action for Health and SMQ, a visual artist who was commissioned to host the workshops [33]. The interpreter assisted at all the workshops ensuring that all verbal communication was translated into the appropriate language, such as instructions for activities and discussions between PPIE contributors and researchers. Information about the project and consent forms were provided in English and Bengali.

The workshops were designed by the research team to be easily accessible and require no previous knowledge of the arts‐based methods. SMQ provided ideas on different arts‐based methods, given their extensive knowledge of visual arts‐based methods and work with diversifying audiences in arts institutions. GC and CD contributed based on their knowledge of the local community and previous PPIE experience whilst ER provided input into the workshops on the different aspects of the wider AI‐Multiply study and how AI is used in healthcare research. ER, GC and CD did not have prior experience working with these arts‐based methods.

To capture discussion from the PPIE contributors over the multiple sessions, we collaborated with a visual minutes artist, Isolde Godfrey from Woven Ink [34]. Visual minutes are a way of live recording discussions through illustrations and words, a popular method used within PPIE work to create accessible visual outputs of discussions [35]. The visual minutes artist created a ‘live’ document, adding new sections during each workshop. This interactive approach allowed the visual minutes artist to listen to discussions, observe sessions and engage with contributors in order to add to the visual minutes. PPIE contributors were encouraged to interact with the artist, sharing their artwork and reflecting on the evolving mural.

### 2.3 Workshops

We conducted a series of six 2‐h workshops over the course of July 2023. At each workshop the PPIE contributors were invited to use different visual art‐based methods (body mapping, collage, ceramics, AI generated images) to explore a particular theme (Table 1). Each session was designed by the research team to prompt creativity and discussion around topics that could influence the broader research project.

**Table 1:** Overview of the contents and methods used in each workshop.

Workshop	Description	Methods
1: Health journey	This workshop explored people's lived experience of MLTCs	Body mapping [36]
2: Medication journey	This workshop was an exploration of people's experience of having multiple medications prescribed	Collage [37]
3: Health service use	This workshop focused on the interaction and contact with healthcare professionals and health services	Collage
4: External factors	This workshop explored the wider factors that impact healthcare such as lifestyle, access to education and languages spoken	Ceramics
5: AI	This workshop focused on the concept of AI and to familiarise contributors with an AI tool	DALL‐E 2 AI generated images [38]
6: Evaluation	The evaluation workshop acted as way to reflecting on what was learnt, what worked well and how PPIE contributors could get involved with other aspects of the AI‐Multiply study. Each of the key priorities identified in previous sessions were displayed visually as leaves on a tree.	Head, heart, bag and bin evaluation [39]
		Tree summary

**Figure 1:**
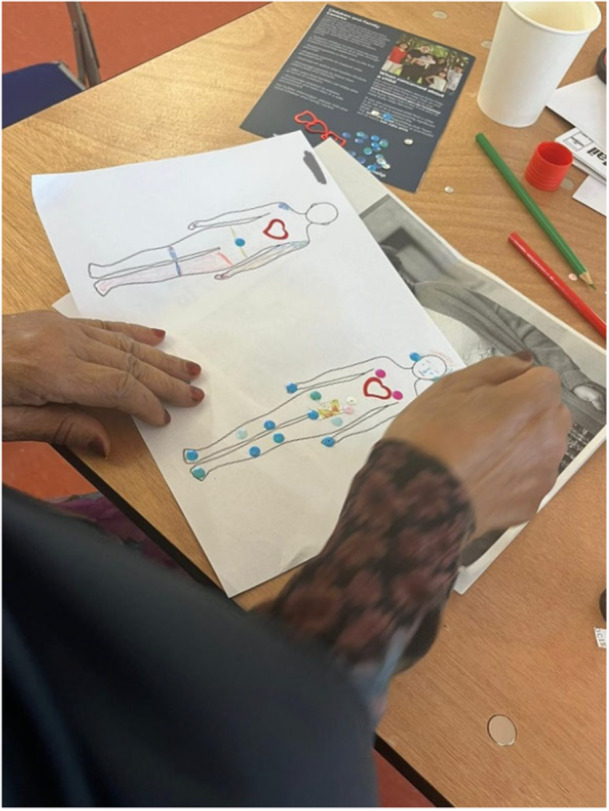
A photo of a participant creating a body map, on the left outline of a body they detailing where they experience unwellness and on the right where they experience wellness.

In the first workshop contributors were asked to use a body mapping technique and to draw in different colours on a 2D print out of a body where they experienced unwellness, and on another body, where they feel well (Figure 1). Body mapping was originally developed to explore individuals experiences in fertility [40] and has since been used as an educational tool, therapeutic intervention and as a participatory research method [41]. It is a reflective process drawing attention to the embodied experience and engaging in knowledge translation [42].

The second and third workshop engaged collage, which involves making a visual representation of a concept using and combining different materials, which could be paper, images, magazines, textiles, photographs, etc. Collage is a visual way to express fragmented experiences and can encourage multiple layers of interpretations due to the nature of the method [37]. Collage has previously been used in healthcare research to map mental health service provision [43] and to aid reminiscence in individuals with dementia enabling information sharing [44]. In the second workshop PPIE contributors explored through collage their experiences of taking multiple medications, and the third workshop focused on healthcare interaction and use.

For the fourth workshop we used clay to explore wider aspects of individuals lives, to prompt a discussion on a more complete picture of health moving away from the biomedical understanding of health. Clay is highly tactile and has been used in research and therapy as a way to explore embodied experiences [45]. It is a highly sensory experience that again doesn't rely on language, or prior ability to use. Contributors were asked to create things out of clay that had been impacted by living with multiple health conditions. They could either then destroy their creation or choose to keep the sculpture.

In the fifth workshop we relied on a generative AI image tool. DALL‐E 2 is a website that uses generative AI to create digital images based on text descriptions (or prompt) provided by the user. DALL‐E 2 was trained on millions of images taken from the internet, and uses these to generate an image that matches the semantic meaning of the text prompt [38]. The use of generative AI systems as a way to generate digital images within research is only just being explored. Generative AI image tools have been used to build visual narratives to support story‐telling [46], and as a way to explore patients experiences and emotions in therapy [47]. There is also growing research about using creative AI formats, such as art installations, that provide a space for a user to interact with an AI system [48, 49]. This ‘*creation, manipulation and sharing of meaning through engaged interaction with artifacts*’ [50] can help to develop AI literacy and make AI more visible and tangible [48].

PPIE contributors were provided with a laptop for the fifth workshop, as well as a predefined log in to reduce the need to enter personal email addressed, and supported by the research team to enter text descriptions into the DALL‐E 2 software on a laptop, either through typing or verbally saying a phrase that one of the research team would type. Contributors were encouraged to create multiple different digital images on any topic they wished. After creating the generated art piece, contributors could then discuss their work with other contributors and the research team. See Supporting Materials for examples of the artwork generated in all of the other workshops.

### 2.4 Evaluation

Throughout the workshops, notes were made by the research team to record discussions with and between PPIE contributors. In particular, notes were captured around PPIE contributors descriptions of their art and visual work and through discussions with the research team this helped to identify potential research priorities or concepts of interest. The PPIE contributors visual work and the visual minutes mural were also used as a way of recording information discussed in each workshop.

**Figure 2:**
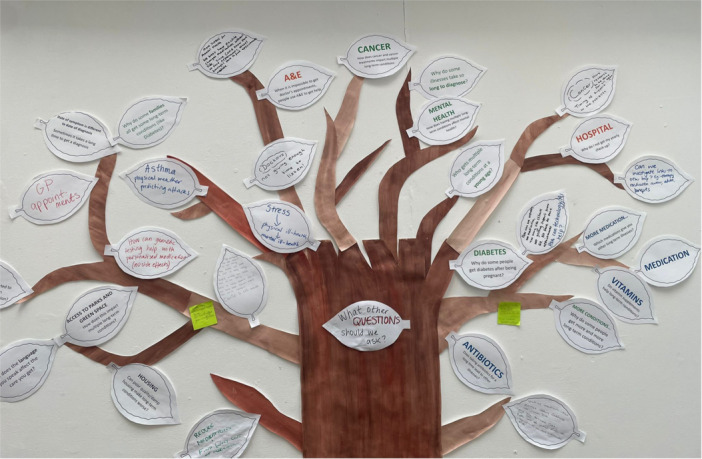
Tree of priorities summarising the discussions, perspectives and views of the PPIE contributors over the workshops.

For the sixth workshop, the research team presented back all the research priorities and concepts discussed by the PPIE contributors over the five workshops. This was in the format of a tree, with each research priority presented on a different leaf (Figure 2). Contributors could walk up and interact with the tree, by adding, adjusting or removing any of the leaves. We also ran a brief evaluation of the workshops, using the established head, heart, bin, bag evaluation method [39]. This is an interactive method uses the outline of a body holding a bag, to capture feedback across four areas. PPIE contributors were provided with pens, paper and stickers and invited to add to the body outline a note to indicate something they learnt (the head), they experienced (the heart), something they would take away with them (the bag) and something they wasn't useful or they didn't like (the bin). Feedback could be written or given in either English or Bengali. Any feedback in Bengali was translated by the interpreter. The direct quotes from PPIE contributors reported in this chapter are taken both from feedback during the workshops, and also from the head, heart, bag, bin exercise. See Supporting Information S1: Materials for an image of the final evaluation.

As a collective group we also developed ideas about how to best share our findings, both the research priorities and also the visual and artwork. We also discussed the best format for this dissemination, for both the wider AI‐Multiply consortium and for the local community. Finally, at the end of the workshops the research team conducted an open structured reflection session, which was audio recorded and transcribed. These reflections alongside the research priorities and evaluation helped to inform this article.

### 2.5 Ethical Approval and Consent to Participate

Ethical approval was obtained from Queen Mary University of London Ethics of Research Committee (QMERC23.127).

The participant information sheet and consent form were made available in English and Bengali, with all documents translated using a professional translation service. One of the research team discussed the participant information sheet and consent form verbally with PPIE contributors, with the assistance of the interpreter when required. Written informed consent for the recording and use of quotes, photographs and artwork was obtained from all individual PPIE contributors. To ensure that photographs only included contributors who provided consent, coloured lanyards were used to help differentiate those who had consented.

## 3 Findings

We reflect on the possibilities and complexities of using visual arts‐based methods to engage a broad range of PPIE contributors in setting the research priorities for AI research.

## 4 Possibilities

### 4.1 Research Priorities

Through using visual image and art‐based methods this facilitated the involvement of individuals whose perspectives are not typically included in AI research. This group of PPIE contributors bought with them their own life experiences of MLTCs, taking multiple medications and their daily knowledge of AI to inform the research priorities for the AI‐Multiply study.

Many of the key research priorities shown in Table 2 reflected the lived experience of the PPIE contributors. The group identified key research areas that were important to them and their communities, this centred around the challenges to get GP appointments and the impact of this on MLTCs and taking multiple medicines. Other core areas were on MLTCs and the role of mental health, early onset MLTCs, Type 2 Diabetes particularly during pregnancy and within families as well as the impact on care for those speak English as a second or additional language.

**Table 2:** List of research priorities and questions that were developed over the six workshops.

**Long‐term conditions** – What is the role of cancer and cancer treatments in the development of MLTCs and polypharmacy? – How does MLTCs and polypharmacy impact mental health? What comes first? – Why do some people get early‐onset MLTC? Who is more likely to get MLTC when they are very young? – What's the role of multigenerational diseases within one family? Why do some families all experience the same combination of long‐term conditions? – Who goes on to get Type 2 Diabetes after gestational diabetes? – Can we use AI to predict periods of health, rather than periods of unwellness? – Why do some people get early‐onset Type 2 Diabetes? – Why do some people get more complications after Type 2 Diabetes? What can we do to stop these complications? – Who is more likely to get complex multimorbidity or more than 2 long‐term conditions? **Medications** – Why role does the time between medication prescriptions play? – How does taking multiple courses of antibiotics impact the onset of MLTCs? – How vitamin supplementation impact MLTCs and polypharmacy? Can this interact with prescribed medication? – Why do some people rapidly get prescribed more medication whilst others are stable? – What medications are interacting and cause further long‐term conditions? – Why are some periods of health associated with a decrease in medication? What can we do to decrease the number of medications someone has to take? **Health service interactions** – As GP appointments are hard to come by many contributors use A&E to seek medical help, is there a way to capture this is the data? – Lack of annual checkups offered by primary care provided and difficulty getting appointments meant that people's long‐term conditions and general health gets worse. What's the connection between who doesn't get offered annual check‐ups and health status? – For those that don't speak English as their first language, or at all, how does this impact how they are perceived by the doctor and getting GP appointments? – Can we use AI to improve getting GP appointments? **Other variables of interest** – Poor quality housing ‐ contributors felt that this was core to many long‐term conditions, and things like poor quality social housing should be included as a measure to understand health – What measures can we include to capture SES or level of deprivation? – What are the employment/work related long‐term conditions? – What is the time between date of first symptom reported versus date of first diagnosis? Is this different for some demographic groups? – Which long‐term conditions or combinations of conditions are most frequently mis diagnosed or diagnosed late? – For those that don't speak English as their first language, or at all, what is the impact on MLTCs and the care received? – Green‐blue space ‐ contributors felt that green or blue spaces were highly beneficial to health and were of interest to this research. Green and blue spaces are natural areas that contain vegetation such as parks (green spaces) or water, such as rivers and the coast (blue spaces).

It is important to note that many of these concepts are not complete or finalised research questions, but areas of interest or further investigation. Some of these research priorities will go on to be used as part of the AI‐Multiply study, this can be in the form of research questions or to identify variables as outcomes for AI models. For example, contributors focused on early‐onset multiple long‐term conditions, and why that is more prevalent in some demographic groups. This can be reframed as an outcome and we can build algorithms to understand who is at risk of early onset multiple long‐term conditions and what factors drive this risk. Contributors also offered a reframing of questions, rather than using AI methods to predict future healthcare problems, contributors were particularly interested in forecasting and understanding periods of wellness. This is a shift away from typical approaches which look at predicting disease, or future drug prescriptions for example.

Other examples of the influence of PPIE on algorithm development are harder to quantify but can help to direct methodological choices. Many of the variables of interest highlighted in Table 2 can be considered when developing AI models in healthcare, for example, poor quality housing was of particular interest to PPIE contributors but is not commonly available within healthcare data. Future research could specifically look to link EHR data to local area level data, such as housing quality, through the English Housing Survey [51].

### 4.2 Visual Art as Communication

Through creating visual arts, rather than relying on focus groups or written materials, we were able to facilitate different forms of communication. Contributors felt that visual art methods gave them a new way to communicate with each other, with researchers and about themselves. One contributor explained *“When I have a body problem sometimes I cannot express it. Here, I can design and express myself”* (P3). The process of creating visual art allowed people to *“express myself through arts without the need to speak”* (P5). For this group of PPIE contributors, the majority of which spoke English as a second, third, or fourth language, found visual art a useful process for communicating. This was for both a way to navigate the language barriers, but also helped contributors communicate concepts about their bodies or health conditions. This resonates with previous research that visual art can help individuals to articulate their health experiences [52].

**Figure 3:**
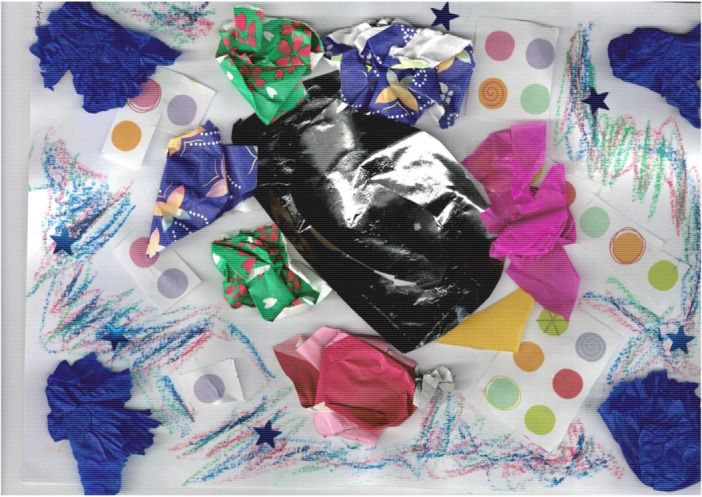
A contributors representation of their experience taking multiple medications is depicted as swirl (shape in black in centre) with increasing numbers of tablets (shown by coloured dots).

**Figure 4:**
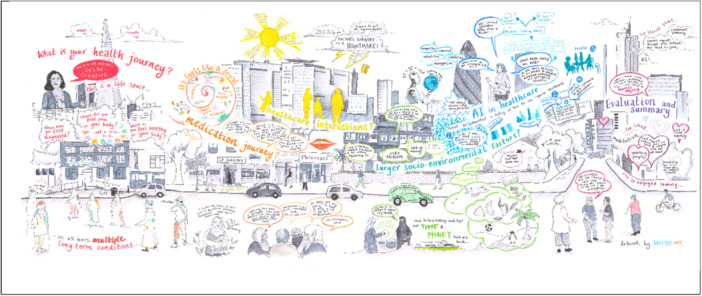
The finished mural, a product of discussions over each workshop. It is possible to see the swirl shown in Figure 3 on the left hand side, next to ‘Medication Journey’.

The mural acted as a live document and a site of communication where contributors and researchers were able to reflect on their experiences, talk to the visual minutes artist and other contributors. Contributors enjoyed seeing their experiences as part of a bigger picture, and to be able to identify themselves, their artwork or comments in the mural. It also worked particularly well to remind everyone what had happened in previous workshops. In Figure 3 there is a collage made by one of the contributors about their experience of taking multiple medications “*The swirl is my timeline, the dots are my pills, I started with 1 and now I take 6*” (P7). In Figure 4, this perspective was incorporated into the larger mural, alongside other discussion points around medication.

**Figure 5:**
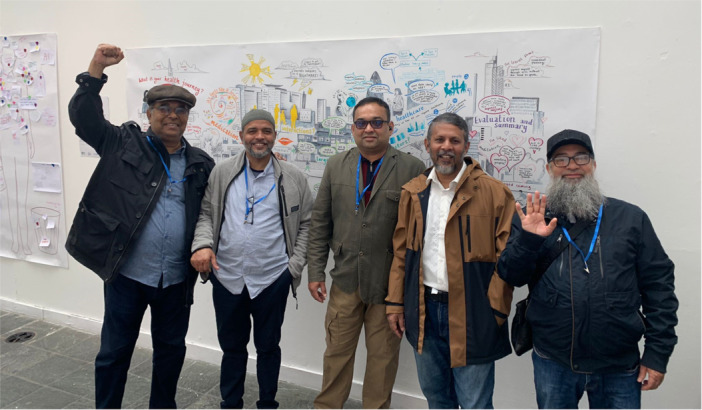
A group of PPIE contributors stood in front of the visual minutes mural at the community art exhibition.

**Figure 6:**
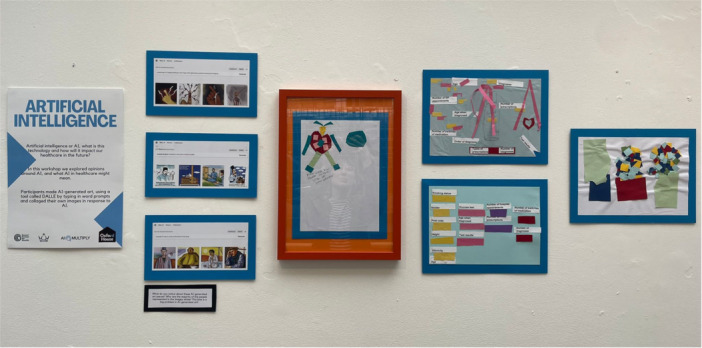
A cluster of art and visual images from the AI workshop on display at the art exhibition.

Finally, the visual art created by the contributors and the mural also served as a form of communication with the wider community and research team. The visual art pieces and mural were shared with the larger AI‐Multiply consortium through an online presentation and via email, which helped to disseminate research priorities and strengthen the importance of PPIE particularly with researchers who were less familiar with this style of working. We also held a local art exhibition in Tower Hamlets where the art pieces, quotes and photos were on display for the local community and wider research community to engage with. Figure 5 shows a collection of some of the PPIE contributors in front of the mural at the exhibition, whilst Figure 6 shows an example of the visual images on display in the exhibition. This was attended by over 80 visitors which helped to promote public involvement and engagement work, and created a space for the PPIE contributors to share their creative pieces with family and friends as well as researchers from the AI‐Multiply project. The artwork and mural continues to be used by our community partner Social Action for Health, but is not currently publicly accessible online.

### 4.3 Making AI Tangible

AI is a complex and tricky subject to talk about, given that it is often intangible and much of the inner workings are hidden. In the AI workshop we attempted to make visible some of the AI pipeline and give contributors hands on interaction with an AI tool.

**Figure 7:**
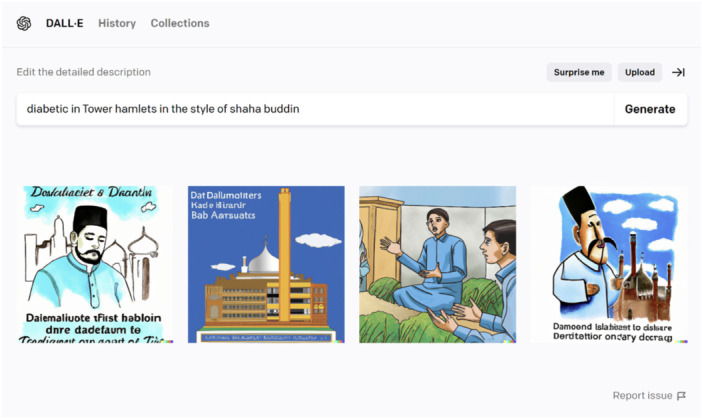
An AI generated art piece, note that the style requested in the prompt of Shahabuddin, a famous Bangladeshi artist, is not reflected in the images generated.

By using DALL‐E 2, an AI system than generates images in response to a text prompt, this helped to make AI more concrete. See Supporting Information S1: Material for a list of different prompts made by contributors. Previous research has highlighted the advantages of using embodied interaction to promote AI literacy [49]. By interacting with an AI system, it also provided a platform to then have conversations around AI and potential benefits and limitations. For example, many of the images generated in the workshops included white individuals in westernised artistic styles, unless contributors used specific text prompts to change this. For example, see Figure 7, where the contributor's text prompt “*diabetic in Tower Hamlets in the style of shaha buddin*”, specifically requests the style of Shahabuddin, a famous Bangladeshi artist, which is not reflected accurately in the images generated. This bias is well‐known and documented in the literature, whereby generative AI systems are predominately trained on western data, reflecting this back in the content generated [53, 54]. This bias was a surprise for contributors, many of who perceived AI to be highly knowledgeable and accurate. This experience highlighted both that AI systems were not always true and that they can contain pre‐existing biases which can lead to discrimination against minoritised groups.

The variety of AI prompts documented through Workshop 5 demonstrate that the group were deeply engaged with medical and health‐related themes, and used DALL‐E as a tool to illustrate these concepts through various artistic styles. This shows an intersection of medical knowledge and lived experience with creative expression, aiming to visualise and communicate health conditions in an accessible and possibly empathetic manner. Within the range of prompts, there is an emphasis on localisation, medical realities and everyday life, where scenarios like a “*Tower Hamlets woman carrying shopping*” are used to contextualise medical conditions within familiar, relatable settings. The prompts are also used to create artistic and anatomical visualisations of diseases like diabetes or fibromyalgia which could have been used for explorative or educational purposes. Interestingly, there was also an exploration of sensations and symptoms, such as depicting tingling sensations with lightning symbols, which aims to communicate symptoms that may be challenging to express verbally, potentially fostering greater empathy and understanding with the viewer. These approaches suggest a strategic use of art to bridge the gap between medical science and personal experiences, with AI as the facilitator of this process.

Finally, discussing AI more generally through arts‐based methods created a space for individuals to air concerns and build additional knowledge around AI. For example, many contributors equated AI to robots, which is widely held belief strengthened by the presentation of AI in both fiction and the news [55]. This was something we were able to discuss as a group, and explore contributors concerns. For many, access to GP appointments was already of major challenge and there were fears that AI and particularly robots would replace GPs and limit the amount of face to face contact that individuals would get with human healthcare providers. There was also excitement in the use of AI, particularly whether it could shift the focus from only focusing on what makes health conditions worse, and instead be used to predict periods of wellness, or factors that can improve health outcomes.

Contributors felt that these workshops helped them to learn about “*AI and new ideas in technology”* (P7) and “*through participating the workshop I feel better as I now have a little understanding of AI*” (P18). By interacting with AI and through discussions this may have enabled contributors to develop different types of AI literacy whilst still being enjoyable. Arts‐based methods may act as a ‘*trojan horse*’, a way to explore complex AI concepts and highlight socio‐technical issues whilst still being fun and accessible [30].

### 4.4 Intrinsic Benefits

Contributors reported enjoying the process of making visual art, ceramics were a particular favourite, and on the social benefits of engaging in research. Contributors felt they were able to make friends and *“meet new people”* (P13), the workshops helped to create a space to get to know others sharing similar health conditions and learn from others experiences. One participant said: “*I was happy that there were other people on the similar understanding. We learned to be happy even though we have so much pain and worries to carry within us.”* Contributors requested more arts and AI sessions in the future and also wanted to invite friends and family members to attend. Art also helped people to feel better *“making art is like therapy”* (P3) which mirrors the rich histories of practice and literature around art therapy [56].

When engaging with this group it was important to ensure that the research benefitted the contributors as well as the research. This frames involvement as a democratic right, which can bring intrinsic value to the contributors and is not just a process to improve the research [57]. These workshops did have positive impacts on contributors that did not directly benefit the research, such as socialising, many contributors in the evaluation reported enjoying ‘*meeting new people*’, feelings of achievement: *“My son said well done Ma!”* (P16) and fun: “*All the six sessions were fantastic. I really enjoyed coming. I got learn a lot of things. I was happy to have to the translator otherwise I would have left the session on the first day*” (P19). During this project there was no drop out in attendance, which is a known problem in PPIE and academic research [58, 59]. We suggest that the fun and social aspects of the workshops may have helped to keep people engaged and prevented drop out, particularly given the high levels of enjoyment in creating art and the development of a social network over the series of six workshops.

There were also additional benefits to the research team as it presented an opportunity to learn and practice PPIE, particularly for ECR researchers, as well as to establish longer term connections with the local community. Particularly, the research team derived a lot of joy from the community art exhibit as it was a creative and fun space to work with the PPIE contributors to display their works.

### 4.5 Collaboration With Community Partners

Previous studies have highlighted the advantages of working with community partners [60], and the underlying success of this project was due to the local community organisation, Social Action for Health. They were able to leverage existing community ties to reach individuals new to research, particularly those from marginalised groups. They invested time regularly communicating with contributors and ensured that contributors were not excluded through typical bureaucratic processes such as completing reimbursement forms. Forms of communication that contributors preferred were also used, such as WhatsApp and phone calls. Considerable work was done behind the scenes to support contributors and make sure they felt comfortable as they participated in something outside of their comfort zones. The interpreter was also vital to the project, contributors liked that the interpreter was consistent throughout the projects and was available at all workshops: “*I have really enjoyed myself. Especially, because we had one translator for our table of four ladies. She translated to us in a way we understood and beyond. I couldn't do this session at all without her support*” (P20).

## 5 Complexities

Many of these complexities faced in this project, such as limited resources, are not new, and are well reported on in the participatory research literature. What this study hopes to add is additional findings around the complexity AI adds.

### 5.1 Communication

There were challenges in ensuring the language used to introduce the research project and AI as appropriate for the group, and easy to translate. Contributors bought their experiential knowledge of AI, which differed greatly, and some contributors did not find the AI workshop engaging. This was particularly exacerbated by the technical challenges, although contributors were supported by both interpreter and researchers to enter in a text prompt into the DALL‐E 2 AI tool, some contributors weren't comfortable using a laptop. For future research we would want to explore other explanation options for how these generative AI systems work, or to use only offline activities, to ensure that everyone felt able to participate.

These workshops helped to start conversations around AI and build some AI literacy, however, many contributors also expressed that this knowledge was limited *“Not a lot people in my community understand what AI is and how it works”* (P8). This emphasises the importance that this is not a one off event, and that involving people for the duration of the research project may be beneficial so they can further develop understanding around AI.

### 5.2 Lack of Resources

Working in this space is compounded by the lack of practical examples and resources available to support this work [61, 62], particularly around easy to understand explanations of AI in multiple languages. As there is a multitude of ways to understand AI, and no clearly agreed on definition, how we can we talk to the public about getting involved in AI research? What are the best ways to communicate about the benefits and limitations of AI? We need more materials, such as [63, 64], which are co‐developed with PPIE contributors to facilitate work in this space and we join others calling for the development of resources to engage with members of the public around AI in healthcare [15, 62].

### 5.3 Existing Structures Limits Participation

Various existing structures impeded the level of participation possible in this project. The overall direction of MLTCs and multiple medicines was already determined by the funding call, and the data available restricted some of the potential avenues of research priorities identified by the PPIE contributors. For example, although contributors identified local pharmacies as playing a key role in MLTC care, this is not available in the data that was available for the AI‐Multiply project. Typically to access public healthcare data there is a long application and ethics process, which can take years to complete and data needs to be applied for prior to research starting. This meant that there needed to be negotiation between the researchers and the PPIE contributors, to manage expectations of what was possible, as was part of the continual process of communication. Due to the open nature of arts‐based research this meant that we identified multiple research priorities which are outside the scope of AI‐Multiply. However, we hope that these can be used to inform future research studies.

In this study, the arts‐based methods and workshops were designed and chosen by the research team, which restricted the involvement of PPIE contributors, future research could explore designing workshops and arts‐based practices in collaboration with PPIE contributors, rather than taking a pragmatic approach.

## 6 Impact on Algorithm Building

As data scientists we exert and embed our own influences, values and perspectives whilst building AI system. The intent is that through participatory research or PPIE activities, we can start to include a wider range of perspectives from patients themselves. Although the researchers on this project are still exploring how we can situate lived experience at the heart of machine learning algorithms and embed this in practice throughout the AI pipeline, we reflect on the ways this work has and will impact our future work.

Most tangibly we will utilise some of the research priorities identified by the contributors, this can be in the form of research questions or to identify variables as outcomes for AI models. For example, contributors focused on early‐onset multiple long‐term conditions, and why that is more prevalent in some communities, such as those in Tower Hamlets. This can be reframed as an outcome and we can build algorithms to understand who is at risk of early onset multiple long‐term conditions and what factors drive this risk. Contributors also offered a reframing of questions, rather than using AI methods to predict future healthcare problems, contributors were particularly interested in forecasting and understanding periods of wellness. This is a shift away from typical approaches which look at predicting disease, or future drug prescriptions for example.

Additionally PPIE contributor perspectives can help shape the evaluation of AI models. PPIE contributors raised concerns about the fairness of different AI models particularly for ethnic minority groups. This can inform evaluation, ensuring that we analyse all models by subgroup performance, looking at ethnic group, sex and indices of multiple deprivation and other demographic metrics.

Other examples of the influence of PPIE on algorithm development are harder to quantify but can help to direct methodological choices. Contributors discussed the impact of missing or incorrect data based on their own experiences of their EHR. Missingness is a well‐studied problem within health data research, and we could specifically use modern deep learning methods that allow for missingness. Deep learning also gives the possibility to include a greater number of variables than traditional epidemiological methods. This may allow interesting opportunities where we can include predictor variables that are important to PPIE contributors, but may not be typically included in models. For example, PPIE contributors often wanted to capture the impact of living in poor quality housing, or the impact of using a interpreter at their GP service on the care received.

These workshops also had an impact on how we talk about and frame AI. PPIE contributors perceptions of AI as robots highlighted the importance of the images and visuals we use to talk about AI, moving forward we will use *Better Images of AI* [65] that move away from stereotyped ‘blue robot’ images. Future work could also be done with the PPIE group to create bespoke visual representations of AI.

Additionally, building on the lack of resources for participatory AI work with patients, and the benefits of developing AI literacy with contributors as part of the process, we plan to run a series of workshops to co‐develop an animation around how we use AI models in research. For PPIE contributors to meaningfully contribute to research in this area, it is important that there are resources to facilitate the understanding of these methods and their limitations.

As this project moves forward we will continue to work with contributors throughout the development of an AI system, across the entire machine learning pipeline, from model development to interpretation of findings and dissemination.

## 7 Conclusion

In this study we employed arts‐based methods to engage a diverse group of PPIE contributors new to research. Collectively over six workshops contributors helped to create a list of research priorities for an AI project on multiple long‐term conditions and multiple medications which went onto inform algorithm building processes.

Both the process of creating visual art and the final pieces facilitated different ways to communicate complex concepts such as multiple long‐term conditions and AI. By interacting with an AI‐generated art tool, this helped to make abstract AI concepts more tangible and accessible, such as the role of bias and the potential for discrimination against minoritised groups. These workshops also provided an opportunity to develop AI literacy and provided a space to start managing expectations of AI.

The visual art pieces and the mural formed sites of communication between the PPIE contributors and the wider AI‐Multiply research team who are engaged in AI algorithm building. By sharing artwork, as well as a final list of research priorities with the wider team, this presented multiple opportunities to understand the perspectives of those with lived experience of MLTCs and multiple medications.

Arts‐based methods also offered intrinsic benefits for both contributors and researchers, such as enjoyment, developing social connections and learning, which in turn benefited the research project through high levels of engagement and no drop out.

Moving forward there is a clear need for more resources and practical examples to support PPIE in AI research, particularly in multiple languages. Additionally, long‐term engagement strategies should be considered to build sustained AI literacy and ensure ongoing input from diverse communities.

## Author Contributions

Conceptualization: Elizabeth Remfry, Sylvia Morgado de Queiroz, AI‐MULTIPLY Consortium, AI‐MULTIPLY PPIE Group, Social Action for Health. Data curation: Elizabeth Remfry, Sylvia Morgado de Queiroz, AI‐MULTIPLY Consortium, AI‐MULTIPLY PPIE Group, Social Action for Health. Investigation: Elizabeth Remfry, Sylvia Morgado de Queiroz, AI‐MULTIPLY Consortium, AI‐MULTIPLY PPIE Group, Social Action for Health. Writing – original draft: Elizabeth Remfry. Writing – review and editing: Elizabeth Remfry, Duncan J. Reynolds, Sylvia Morgado de Queiroz, Michael R. Barnes, Rohini Mathur, AI‐MULTIPLY Consortium, AI‐MULTIPLY PPIE Group, Social Action for Health. Supervision: Michael R. Barnes, Rohini Mathur. Funding acquisition: Michael R. Barnes, Elizabeth Remfry, AI‐MULTIPLY Consortium, Social Action for Health.

## Ethics Statement

Ethical approval was given from Queen Mary Ethics of Research Committee (QMERC23.127). Informed consent was obtained from all individual PPIE contributors.

## Consent

Consent for the inclusion of photographs and individual artwork was sought during the informed consent from all PPIE contributors and followed Social Action for Health policies.

## Conflicts of Interest

The authors declare no conflicts of interest.

## Supporting information


**Figure 1:** Two body outlines decorated by a contributor. On the left is the experience of unwellness, and on the right is wellness. **Figure 2:** This art work demonstrates the contributors experiences of taking multiple medications. **Figure 3:** This photo shows the materials spread out for collage, there is a table with pens, magazines, newspapers, pens, glue and other materials. **Figure 4:** In this photo, a contributor is pointing out the ceramic pieces they made, there are several items of food made from clay, as well as a palm tree. **Figure 5:** This is an AI generated image that depicts a pancreas in different styles. **Figure 6:** There is an outline of a body holding a bag, there are many pieces of paper with coloured writing stuck around the outline of the body.

## Data Availability

Data sharing is not applicable to this article as no datasets were generated or analysed during the current study.
