# Supplementary material for: Using Arts‐Based Methods to Involve People Living in Tower Hamlets With Multiple Long‐Term Conditions in the Development of Artificial Intelligence Tools in Healthcare Research
Source: Health Expect. 2026 Mar 1;29(2):e70621. doi: 10.1111/hex.70621 (PMC12950819; doi:10.1111/hex.70621)
Supplement: Supplementary file 1 — Figure 1: Two body outlines decorated by a contributor. On the left is the experience of unwellness, and on the right is wellness. Figure 2: This art work demonstrates the contributors experiences of taking multiple medications. Figure 3: This photo shows the materials spread out for collage, there is a table with pens, magazines, newspapers, pens, glue and other materials. Figure 4: In this photo, a contributor is pointing out the ceramic pieces they made, there are several items of food made from clay, as well as a palm tree. Figure 5: This is an AI generated image that depicts a pancreas in different styles. Figure 6: There is an outline of a body holding a bag, there are many pieces of paper with coloured writing stuck around the outline of the body. [file HEX-29-e70621-s001.docx]

**Supplementary Materials**

**Using arts-based methods to involve people living in Tower Hamlets with multiple long-term conditions in the development of artificial intelligence tools in healthcare research**

**Contents:**

Page 2: Workshop 1: Body Mapping

Page 3: Workshop 2: Collage

Page 4: Workshop 3: Collage

Page 5: Workshop 4: Clay

Page 6: AI generated images and contributor prompts

Page 8: Head, heart, bag, bin evaluation

**Workshop 1: Body Mapping**

| **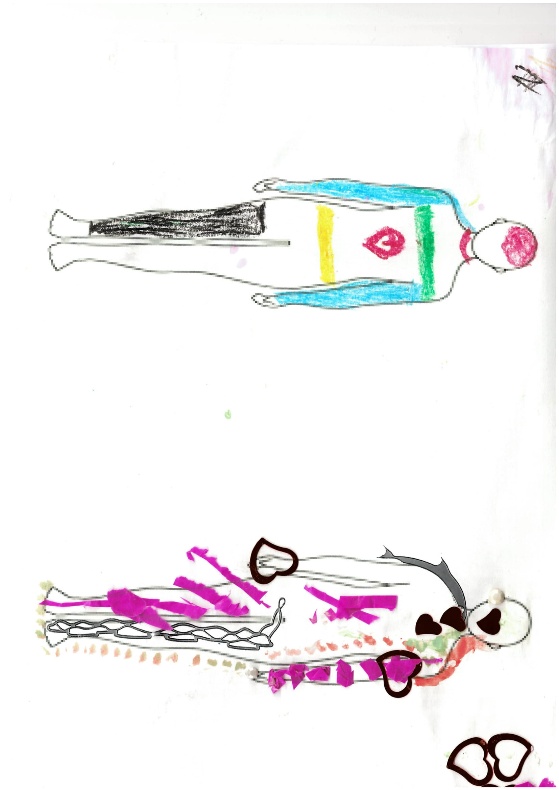** |
| --- |
| Figure 1. Two body outlines decorated by a contributor. On the left is the experience of unwellness, and on the right is wellness. |

This image shows a two bodies, on the left is the unwell body and on the right shows wellness. This contributor found it challenging to describe what living with Type 2 Diabetes felt like. After discussion with a small group of other PPIE contributors who all shared the same health condition, they expressed that they experienced it in their legs. They still wanted to demonstrate that there were many areas of their body where they felt well, as shown in the body on the right. These art works also highlighted to the research team the complexity of multiple long-term conditions, and the multiple body systems that can be involved when experiencing MLTCs. Often symptoms such as tingling in the legs are associated with diabetic neuropathy.

**Workshop 2: Collage**

| 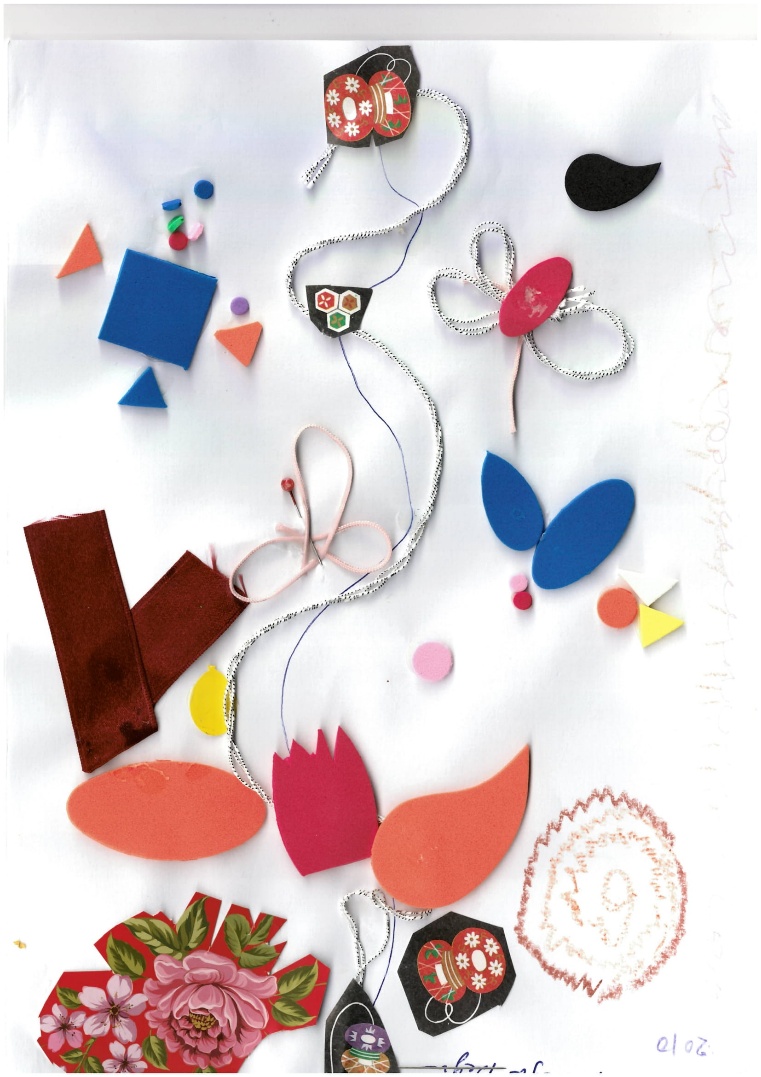 |
| --- |
| Figure 2. This art work demonstrates the contributors experiences of taking multiple medications. There is a white background with different coloured shapes, as well as ribbons and textures |

This contributor represented their experiences of taking multiple medications over their life. They described the art as: *“I am happy with my medication, I do a lot of exercise, all the things in the picture are positive, but the black and blue shapes are when I am feeling down, and the blue squares and dots represent when I don’t get an appointment at the doctors, I feel messy” (P11)*

This helped reveal some of the different aspects of care and managing MLTCs that went on to become some of the different research priorities.

**Workshop 3: Collage**

| **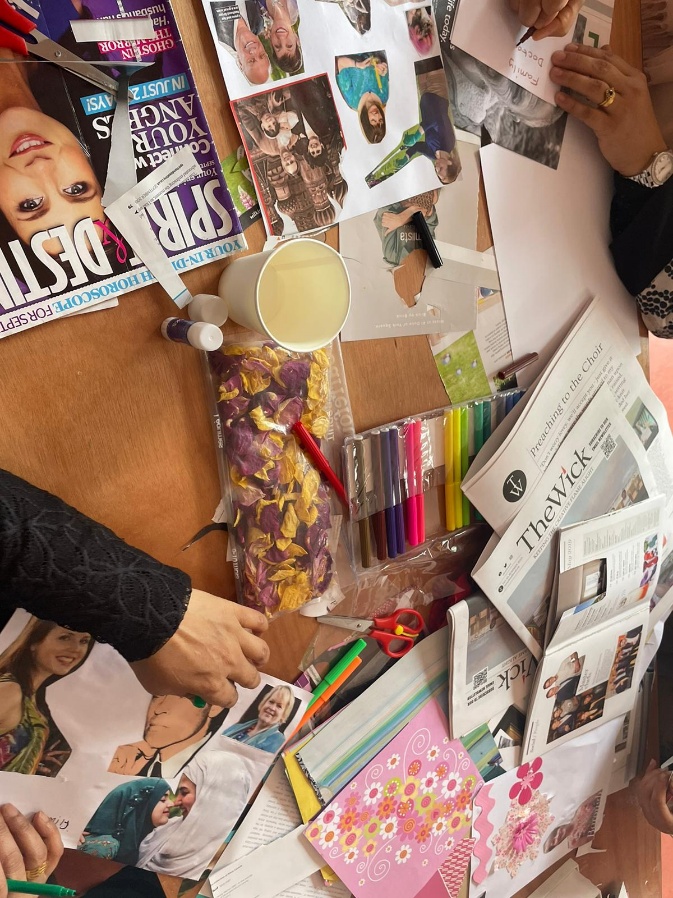** |
| --- |
| Figure 3. This photo shows the materials spread out for collage, there is a table with pens, magazines, newspapers, pens, glue and other materials |

This is a photo taken during Workshop 3 during the collage session. Magazines, newspapers, pens, glue, stickers, coloured paper and other materials were freely available to use. The stickers were particularly a favourite with the group.

**Workshop 4: Clay**

| 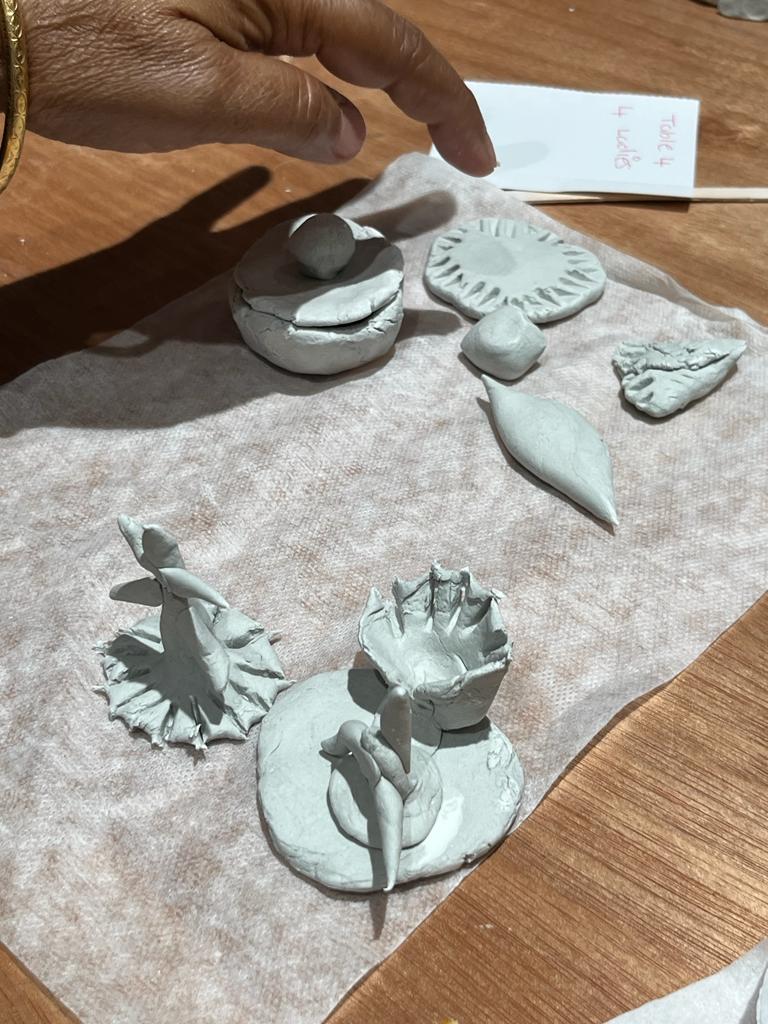 |
| --- |
| Figure 4. In this photo, a contributor is pointing out the ceramic pieces they made, there are several items of food made from clay, as well as a palm tree |

This contributor made various pieces out of clay that represented cooking, which was something they loved, and food that was important to them (see top of the photo for clay samosas and roti). They had had to change their eating habits and customs after getting diagnosed with several long-term conditions.

They also spoke about the difficulties travelling now, and not being able to go on holiday or visit family.

**Workshop 5: AI generated images**

| **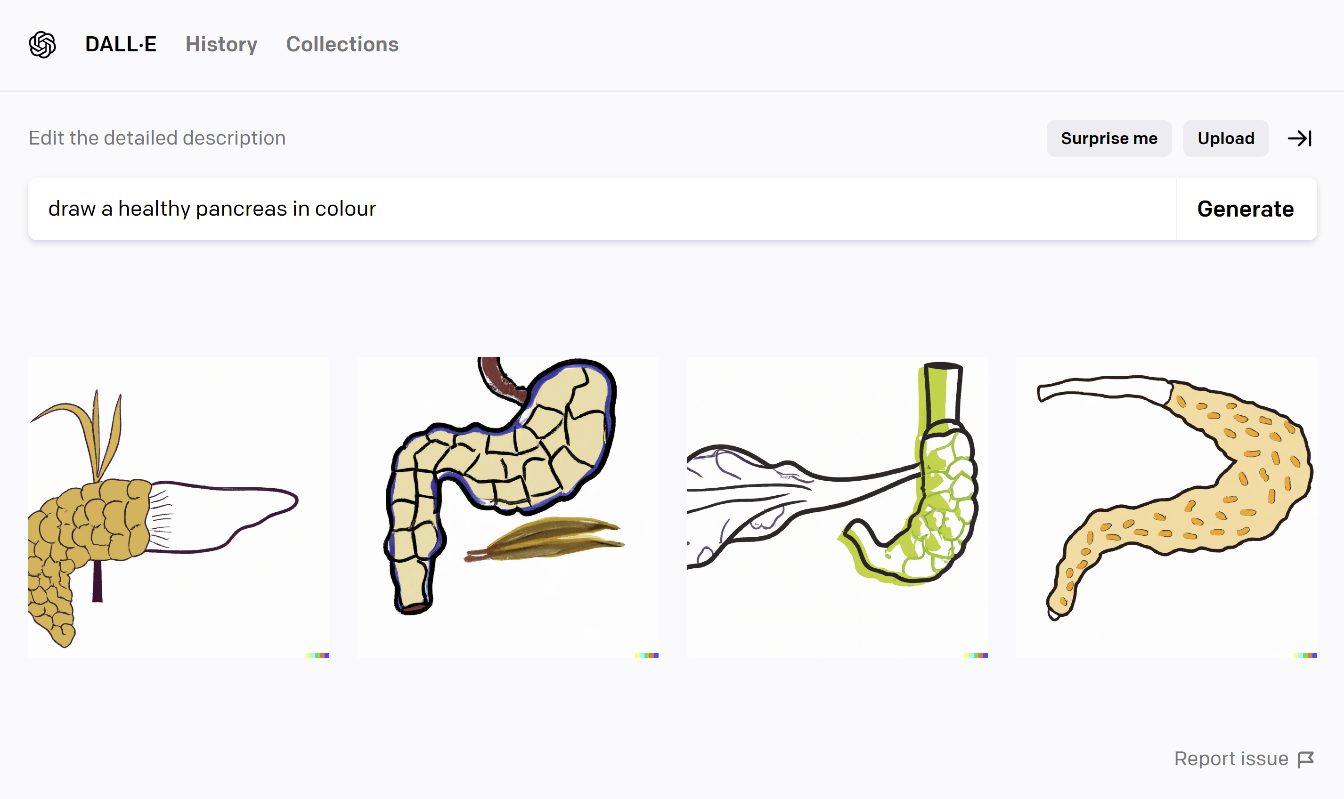** |
| --- |
| Figure 5. This is an AI generated image that depicts a pancreas in different styles |

This visual image was produced using the generative AI image tool, DALLE-2. The contributors prompt is shown above in the description box: “*draw a healthy pancreas in colour*”. Contributors were encouraged to enter their own prompts and request for additional changes to the images produced. This visual piece prompted a discussion around how we can talk about what Type 2 Diabetes feels like, as it often is hard to describe and might not be visible to others.

Other prompts for the generative AI tool, please note these have been copied exactly as they were written and entered:

- “Tower hamlets woman carrying shooping in the style of a photo”
- “Hayfever tablets rainbow coloured”
- “draw a healthy pancreas in colour”
- “heart disease in 1920”
- “a man going to a GP surgery to find out about his cholesterol in the style of monet”
- “a picture of what the pancreas does when you have diabetes type 2”
- “mentally ill man in crisis in the style of van goug”
- “a picture of a stroke victim in the style of dali”
- “fibromyalgia arthritis in body system”
- “good hearted person”
- “blood test diabetic person in the style of a bright picture”
- “xray of a sore shoulder in the style of andy warhol”
- “an 80s photograph showing people talking about AI”
- “type 2 diabetic 60 year old people”
- “a painting of a tingling feeling in the finger with lightening symbols showing the tingling”

**Workshop 6: Head, heart, bag, bin**

| **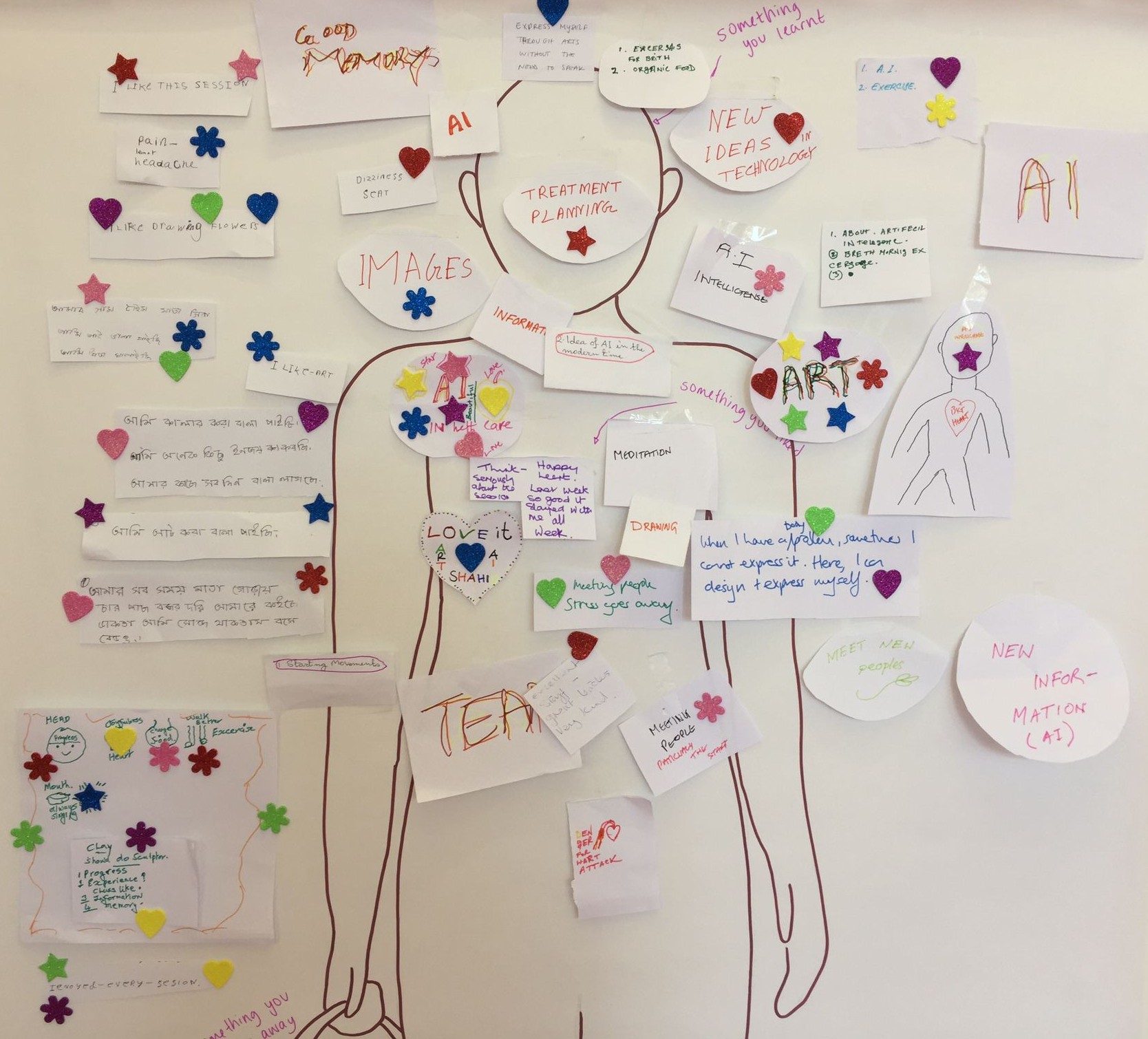** |
| --- |
| Figure 6. There is an outline of a body holding a bag, there are many pieces of paper with coloured writing stuck around the outline of the body |

This image is of part of the evaluation exercise, head, heart, bag, bin. Contributors could write on pieces of paper, on the human outline, or work with one of the research team to write down something they liked, that they would take away, that they disliked and that they had learnt.
